# 50-nm-resolution full-field X-ray microscope without chromatic aberration using total-reflection imaging mirrors

**DOI:** 10.1038/srep46358

**Published:** 2017-04-13

**Authors:** Satoshi Matsuyama, Shuhei Yasuda, Jumpei Yamada, Hiromi Okada, Yoshiki Kohmura, Makina Yabashi, Tetsuya Ishikawa, Kazuto Yamauchi

**Affiliations:** 1 Department of Precision Science and Technology, Graduate School of Engineering, Osaka University, 2-1 Yamada-oka, Suita, Osaka 565-0871, Japan; 2 JTEC Corporation, 2-4-35, Saito-Yamabuki, Ibaraki, Osaka 567-0086, Japan; 3 RIKEN SPring-8 Center, 1-1-1 Kouto, Sayo, Hyogo 679-5198, Japan; 4 Center for Ultra-Precision Science and Technology, Graduate School of Engineering, Osaka University, 2-1 Yamada-oka, Suita, Osaka 565-0871, Japan

## Abstract

X-ray spectromicroscopy with a full-field imaging technique is a powerful method for chemical analysis of heterogeneous complex materials with a nano-scale spatial resolution. For imaging optics, an X-ray reflective optical system has excellent capabilities with highly efficient, achromatic, and long-working-distance properties. An advanced Kirkpatrick–Baez geometry that combines four independent mirrors with elliptic and hyperbolic shapes in both horizontal and vertical directions was developed for this purpose, although the complexity of the system has a limited applicable range. Here, we present an optical system consisting of two monolithic imaging mirrors. Elliptic and hyperbolic shapes were formed on a single substrate to achieve both high resolution and sufficient stability. The mirrors were finished with a ~1-nm shape accuracy using elastic emission machining. The performance was tested at SPring-8 with a photon energy of approximately 10 keV. We could clearly resolve 50-nm features in a Siemens star without chromatic aberration and with high stability over 20 h. We applied this system to X-ray absorption fine structure spectromicroscopy and identified elements and chemical states in specimens of zinc and tungsten micron-size particles.

The renowned discovery by Robert Hooke of the microstructure within cork, which he first termed the ‘cell’, was achieved by employing his state-of-the-art microscope in the mid 1600s^1^. Since then, advancements in microscopes have provided novel insights in a wide range of scientific fields while significantly contributing to scientific progress. Advancements in microscopes have been more recently extended to enabling the viewing of objects on a nanoscale and objects that are invisible using traditional instruments. An important development in these advancements is the X-ray microscope. The utilization of X-rays, which are electromagnetic waves with much shorter wavelengths than visible light, has enabled the use of high-resolution microscopes. For example, state-of-the-art X-ray microscopes^2^,^3^ and focusing optics^4^,^5^,^6^,^7^,^8^ have achieved highly refined resolutions and spot sizes. Specifically, a 3-nm resolution^2^ and 5-nm spot size^8^ were reported. In addition, a high penetration capability of X-rays into an object makes it possible to observe object interiors, even under various environments (e.g. in aqueous solutions and gaseous atmospheres), which cannot be observed by transmission electron microscopes.

Another significant advantage of the X-ray microscope is its capability of analysing chemical states in objects while visualizing the structures, i.e., spectromicroscopy. For example, full-field X-ray absorption near-edge structure (XANES) imaging^9^, which is a renowned type of X-ray absorption spectromicroscopy, can provide chemical mapping in the nanoscale range, especially information of oxidation states and site symmetries. The key element for this microscopy type is the imaging optical device. To date, most developed X-ray microscopes utilize the Fresnel zone plate (FZP) because of its obtainable high spatial resolution. Currently, spatial resolutions (half-periods) of 10–20 nm have been reported in the soft and hard X-ray regions^10^,^11^. However, FZPs have strong chromatic aberrations, depending on the total number of zones; thus, they can handle only highly monochromatised X-rays^12^. No substantially available imaging optical devices exist that can provide achromatic and high-resolution image formation for spectromicroscopy.

A possible approach to addressing the chromatic aberration is use of imaging optics based on total reflection. In our previous studies, development of an achromatic X-ray microscope based on four total-reflection mirrors (i.e. advanced Kirkpatrick–Baez (KB) mirror optics) was attempted^13^,^14^. Advanced KB mirror optics has the advantages of high efficiency and long working distance as well as an achromatic imaging. These unique features are required for practical applications, where detection of very weak signals and careful environmental control are usually undertaken. Thus far, the spatial resolution of 100 nm without chromatic aberration was achieved for the first time owing to ultra-precisely fabricated mirrors^15^. However, the high degree of freedom resulting from the four separated mirrors makes the mirror alignment difficult to achieve and unstable. This optical system can realize a sub-50-nm resolution together with no chromatic aberration; nevertheless, the apparent limitations prevent the achievement of the resolutions and applications required for practical studies.

To address the above issues, this paper presents the development of an imaging optical system based on two monolithic imaging mirrors^16^ (see Fig. 1). The mirrors have elliptic and hyperbolic shapes, respectively, on a single substrate. The fixation of the relative position between the ellipse and hyperbola, which is very sensitive to the image quality, can provide long-term stability and effective usability. Nonetheless, fabrication of such a complex mirror is more difficult than that of general elliptical or hyperbolic mirrors. Using a modified fabrication procedure, the proposed mirrors were completed with sufficiently good shape and an accuracy of ~1 nm, which can avoid the influence on the wavefront.

Tests for spatial resolution, chromatic aberration, and long-term stability were performed using a fine test pattern at BL29XUL^17^ of SPring-8 to demonstrate the performance of the constructed microscope. High-resolution XANES spectromicroscopy visualized distributions of elements and chemical states, showing the feasibility of practical applications of the proposed system. According to the results, the developed microscope provided a spatial resolution of 50 nm without chromatic aberration and a stable observation for more than 20 h.

## Results

### Advanced KB mirror system with two monolithic imaging mirrors

In this study, two monolithic imaging mirrors, which follow Wolter-type I optics^18^, were designed to realize X-ray imaging with high magnification and a large numerical aperture (NA) (Table 1). The wave-optical calculation based on the Fresnel–Kirchhoff integrals^19^ revealed that the achievable field of view (FOV) is 22.9 (11.3) μm in the vertical (horizontal) direction. The full width at half maximum (FWHM) of the point spread function (PSF) at the centre of the FOV is 38 nm (36 nm) at an X-ray energy of 10 keV. Reflectivity after the quadruple reflections using rhodium coating is ~58% at 10 keV, which can provide an X-ray up to ~12 keV (quadruple reflectivity = ~32%).

Despite the above achievements, the solution employing the monolithic imaging mirrors presents a new challenge: the complex mirror shape must be fabricated with high accuracy. On account of this requirement, a solution has rarely been attempted. The entire shape, which appears as a steeply curved ‘V’, along with each aspherical shape, must be simultaneously and precisely fabricated. Our estimation using the respective wave-optical and ray-tracing simulations produced severe tolerance errors for the relative positioning of the two surfaces in the vertical and horizontal directions (see Fig. 2). Furthermore, the required shape accuracy for each aspherical shape was simply estimated using Bragg’s law^4^. When it was most strictly estimated, the required shape accuracy was 1.5 nm, which was twice as stringent as that of the equivalent KB mirrors. This is because the wavefront errors accumulated after each reflection. This means that each mirror had to be fabricated with a shape accuracy of λ/8.

**Figure 1:**
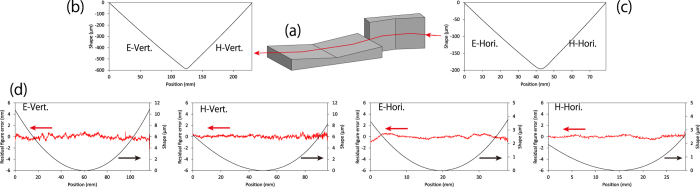
Advanced Kirkpatrick–Baez (KB) mirror optics based on two monolithic mirrors. (**a**) Mirror arrangement. (**b**,**c**) Whole mirror shapes. (**d**) Shapes and residual shape errors on the each section. ‘E-’ and ‘H-’ respectively represent the ellipse and hyperbola. ‘Vert.’ and ‘Hori.’ represent vertical and horizontal directions, respectively.

**Figure 2:**
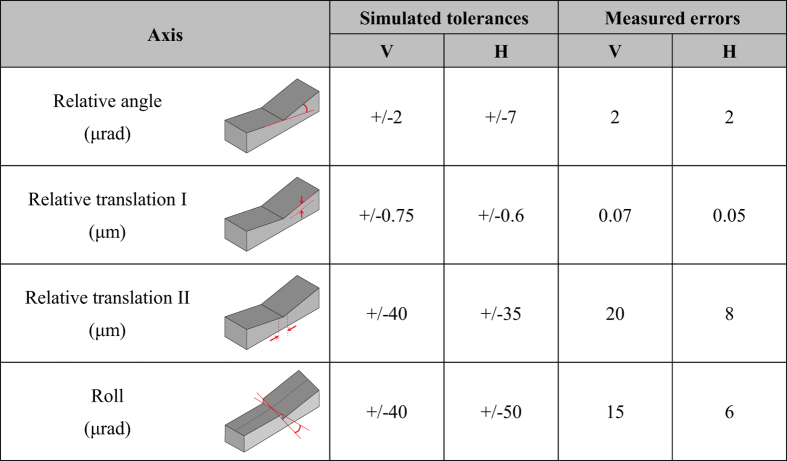
Simulated tolerances of the shape errors and actually measured errors. ‘V’ and ‘H’ denote the mirrors in vertical and horizontal directions, respectively.

We successfully fabricated the two mirrors to satisfy these strict requirements using computer-controlled elastic emission machining (EEM)^20^, a Michelson-type white light interferometer, a Fizeau-type interferometer, and a point-autofocus-type coordinate measuring machine^21^ (see the Method section). The shape accuracy for each ~1-nm surface (see Fig. 1) was achieved in addition to sound accuracies of the whole shape (see Fig. 2). The actual wave-optical simulation considering the manufacturing errors additionally showed that the errors were negligible for obtaining an ideal resolution.

The two mirrors were assembled in a crossed geometry—the same type used in the KB geometry—using a specially developed alignment system. The remaining major degree of freedom was the roll between the two mirrors along the optical axis. The mirrors were easily adjusted with adequate accuracy of ~10 μrad in the same way as in the KB system. This adjustment was performed using two autocollimators and a pentaprism^22^. The full mirror positioning was completed with satisfactory accuracies.

### Full-field X-ray microscope system

**Figure 3:**
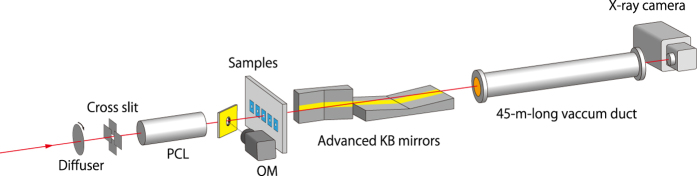
Experimental setup of the microscope system.

A microscope system was constructed for the performance test at SPring-8 (Fig. 3). The X-rays emitted from the undulator at BL29XUL were monochromatised to ΔE/E of 0.013% (approximately 10 keV) using an Si 111 double-crystal monochromator (DCM). A rotating diffuser was placed at the highest upstream point of the first experimental hutch to reduce unwanted interference. A polycapillary lens (PCL; Hamamatsu Photonics) with a focal length of 45 mm and a diameter of 4 mm was used to moderately collect X-rays at the sample position as a condenser. It was additionally used as a deflector by employing the outermost region. This is because the imaging mirrors significantly inclined the X-ray trajectory by 20 mrad, making it difficult to deliver X-rays to the camera placed 45 m downstream of the imaging mirrors. The tilted trajectory was compensated by deflecting in advance the X-ray path with the PCL by the equal but opposite angle. A slit placed just upstream of the PCL was used to block the X-ray reflections, except on the effective region of the mirrors. Thus, the condenser had a nearly equal NA to one of the objectives.

The sample was mounted on the sample holder with XYZ stages. It could be observed with an optical microscope (OM) for matching the region of interest and the X-ray illumination region. The X-rays passing through the 45-m-long vacuum duct after reflecting on the imaging mirrors were recorded with an X-ray camera (AA20MOD +ORCA-Flash 4.0, Hamamatsu Photonics). The camera system consisted of a thin scintillator (P43, thickness of 10 μm), a lens (×2.1), and a complementary metal-oxide semiconductor (CMOS) camera sensor (2048 × 2048 pixels; 6.5 μm/pixel). The effective pixel size was 3.1 μm at the camera, which corresponded to 15.8 nm (4.9 nm) in the vertical (horizontal) directions at the sample position.

### Imaging tests

**Figure 4:**
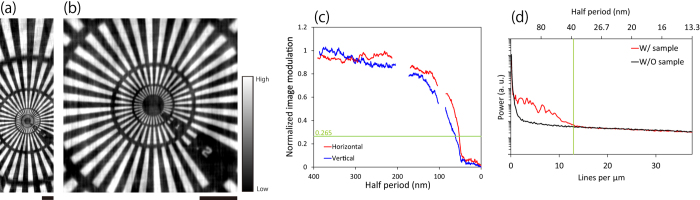
Bright-field X-ray image of (**a**) whole image and (**b**) magnified image. Results of analysis with (**c**) contrast analysis and (**d**) PSA. Exposure = 500 s. X-ray energy = 9.881 keV. Bar = 2 μm.

A Siemens star pattern (XRESO-50HC, NTT Advanced Technology Corporation)—which was made of tantalum (Ta) with a 50-nm feature at the innermost region and a thickness of 500 nm—was used to demonstrate the spatial resolution of the optical imaging system. First, to increase the absolute contrast, the X-ray energy was set to 9.881 keV, which corresponded to the Ta L_3_ absorption edge. Figure 4(a) show the obtained X-ray bright field image. Additionally, images with different exposures of 10 s and 60 s are shown in Supplementary Fig. S1. The 50-nm feature at the innermost region is clearly visualized.

To estimate the achieved resolution, a contrast analysis to investigate the image contrast of periodic patterns was performed (see the Method section). Figure 4(c) shows the relationship between the normalized image modulation and the spatial frequency. In Rayleigh’s spatial resolution criterion^23^, which is one of the most severe criteria, the minimum spatial resolution is defined as the minimum distance between two points resolved with a normalized contrast of 26.5%^23^. Based on the criteria, the achieved spatial resolution was 61 nm (52 nm) in the vertical (horizontal) directions, respectively.

Furthermore, power spectrum analysis (PSA)^23^,^24^ was performed. PSA is a method of estimating the smallest detectable features (see the Method section). Consequently, the PSA revealed that the image had information of a spatial frequency up to 13 lines/μm, which corresponded to a half period of 38 nm (see Fig. 4(d)).

**Figure 5:**
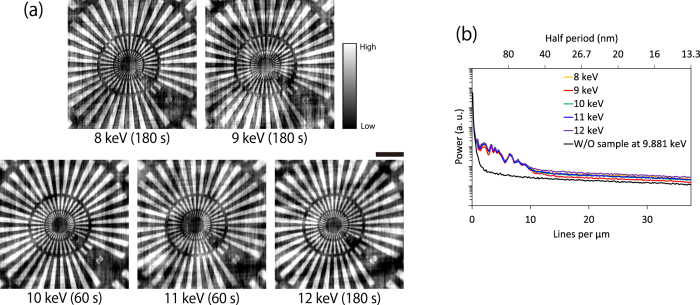
X-ray energy dependence between 8 and 12 keV. (**a**) Bright-field X-ray images. (**b**) Results of PSA. Exposure is shown below each image. Bar = 2 μm.

Next, wavelength dependence was investigated by performing the same tests while changing the X-ray energy between 8 and 12 keV. The obtained images and corresponding PSA results showed no image degradation depending on the X-ray energy (see Fig. 5). However, the signal level on the high-frequency range changed, thereby demonstrating a different S/N ratio due to the different amounts of X-rays arriving at the camera. This was caused by the dependence of reflectivity and air absorption on the X-ray energy. The X-ray signal loss could easily be reduced by changing the mirror design and eliminating the air path. Thus, it was confirmed that the optical system could provide achromatic X-ray images.

**Figure 6:**
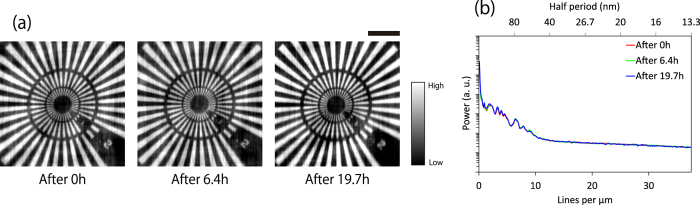
Long-term stability. (**a**) Time-lapse bright-field X-ray images. (**b**) Results of PSA. Exposure = 60 s. X-ray energy = 9.881 keV. Bar = 2 μm.

In addition, a test to evaluate the long-term stability was performed. Three images were obtained under the same conditions without readjusting the optical system after the fine adjustment at 6.4 h and 19.7 h (see Fig. 6). The image quality and PSA results did not change, although the temperature near the mirror changed by 0.3 K during the test, which was not actively controlled. The result showed that the monolithic structure helped well stabilize the optical system.

### High-resolution XAFS spectromicroscopy

**Figure 7:**
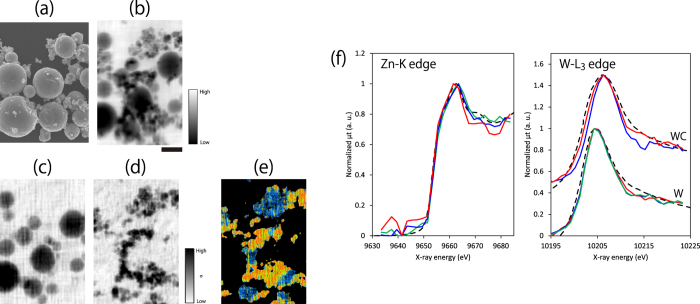
(**a**) SEM image. (**b**) X-ray image averaged over XAFS images between 10,159 and 10,655 eV, showing the existence of Zn and W particles. (**c**,**d**) Distributions of standard deviation (σ) of a series of XAFS images, showing the drastically changing area for image contrast during the XAFS measurement, i.e. Zn and W distributions, respectively. (**e**) Peak-shift map to identify W and WC. (**f**) XAFS spectra averaged over a 100 × 100 nm^2^ square area. Energy scan: (**c**) 9640–9690 eV every 2 eV, and (**d**) 10195–10225 eV every 1 eV. (**e**) Red and blue regions represent W and WC, respectively. (**f**) Solid lines represent the obtained spectra on the different particles. Dash lines represent the reference spectra, which were obtained from the XAFS database (Institute for Catalyst, Hokkaido University) (data information: sample = Zn foil; correspondence = Kiyotake Asakura; date = 2006.12.13) for Zn, and the article (Fig. 2) published by Uo *et al*.^25^ for W. All images were obtained with an exposure of 60 s. Bar = 2 μm.

XAFS imaging was performed to check the practicality of the microscope. The employed samples were particles of pure zinc (Zn), pure tungsten (W), and tungsten carbide (WC), which were spread on a thin SiN membrane at a thickness of 270 nm. The used condition was the same as those of the above imaging tests. A series of images were recorded while changing the X-ray energy in the range of a few tens of eV across the absorption edges of the W L_3_ edge and the Zn K edge. The obtained XAFS images exhibited a drastic change of the absorption distributions, which were consistent with the well-known XAFS spectra of Zn and W^25^ (Fig. 7). The obtained spectra at each pixel enabled the elemental identification of Zn and W. Moreover, some particles with a slight edge shift corresponding to WC could be found from the peak-shift map and spectra (Fig. 7(e,f)). Thus, these results demonstrated that the imaging system can be employed for actual experiments, such as with XAFS imaging.

## Discussion

**Figure 8:**
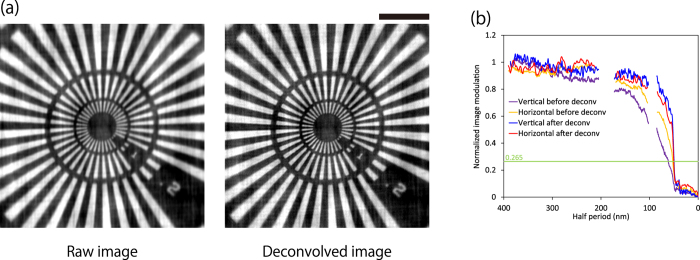
(**a**) Bright-field X-ray images before and after correction using deconvolution processing. (**b**) Result of PSA. The image was deconvoluted with a Gaussian with FWHM of 73 nm (36 nm) in the vertical (horizontal) direction. Bar = 2 μm.

In all imaging results, a difference of spatial resolution was apparent between the vertical and horizontal directions. This resulted from the blurring by the employed camera. The FWHM of the blurring was experimentally estimated to be 14 μm, which corresponded to 71 nm (22 nm) in the vertical (horizontal) directions, respectively, at the sample position with consideration of the magnification factors. The blurring was highly systematic; thus, they could be easily eliminated with the standard deconvolution algorithm. The image was deconvoluted with a two-dimensional Gaussian with FWHMs of 73 nm (36 nm) in the vertical (horizontal) direction (Fig. 8). Although the FWHMs were determined by a trial and error approach, they were very consistent with the camera blurring. The additional contrast analysis after the deconvolution process revealed that the spatial resolutions provided by the developed optical system were 49.6 nm (50.2 nm) in the vertical (horizontal) direction. However, the estimation may have been affected by the Siemens star because the minimum feature of the pattern was 50 nm. As shown by the rapidly dropping curve at the 50-nm half period in the graph, the result cannot provide an accurate resolution limit. The real resolution seems to have been slightly better than the results. The theoretically achievable minimum resolution considering the NA was 23 ~ 29 nm at 9.881 keV, which depended on the degree of coherence of the employed illumination^10^. By using a more high-resolution X-ray camera with minimal blurring and finer test patterns with a 10-nm feature and at least a 5-nm edge sharpness—e.g. the cross section of a multilayer film^10^—a significantly improved adjustment for the focal length and FOV could have been achieved. By overcoming other minor problems, such as the thermal drift of the sample and the imperfection of the condenser, we can approach the theoretical limit in the near future.

The developed achromatic imaging optical system can provide a resolution of at least 50 nm with effective usability and long-term stability. This system is highly promising for various applications, even those using polychromatic X-rays, such as ultra-fast imaging with non-monochromatised intense X-rays^26^, imaging of X-ray fluorescence^27^,^28^, and laboratory-based X-ray applications. Especially, the study of high-resolution full-field X-ray fluorescence imaging remains virtually untouched. Moreover, the imaging mirror will be very useful for many applications, even focusing optics. A general focusing mirror with only an elliptical shape is known to be very sensitive to misalignment of the grazing-incidence angle^29^, resulting in poor resolution and frequent troublesome readjustments of the focusing system. This is because the focusing mirrors suffer from the comatic aberration. However, the imaging mirror can overcome the misalignment problem, even when the error of the grazing-incidence angle is +/−80 μrad, as in our case (horizontal direction), because it can cancel the error by the double reflection. Thus, this type of imaging mirror system will be a powerful tool for focusing optics as well as for imaging optics in the fields of synchrotron radiation X-rays and X-ray-free electron lasers^30^,^31^.

## Methods

### Mirror fabrication

The mirrors were fabricated on synthetic silica substrates by numerically controlled elastic emission machining (NC-EEM)^20^. NC-EEM can produce an arbitrary shape by deterministically controlling the scanning speed of the nozzle head to feed slurry based on the shape data. In our study, the shape errors were precisely measured using a microstitching interferometer (MSI)^32^, which can measure the shape with a high resolution of 35 μm/pixel; however, it is not reliable in a low spatial frequency range. In addition, they were precisely measured using a relative angle determinable stitching interferometer (RADSI)^33^, which can measure a low-spatial-frequency shape with 1-nm shape accuracy, and a point-autofocus-type coordinate measuring machine^21^, which can easily measure the steeply curved whole shape with a 10-nm accuracy. These shape data were precisely combined into one shape data, considering the reliable spatial frequency range of each instrument. The data was input into the NC-EEM system for deterministic shaping. The shaping was repeated several times until the required shape accuracy was obtained.

The processed surface after finishing the mirrors was characterized using a microscopic interferometer (Zygo, NewView 200CHR). Consequently, a root-mean-square roughness of better than 0.2 nm over an area of 64 × 48 μm^2^ (effective pixel size = 200 nm) was obtained, which was sufficiently small to enable us to ignore the reflectivity degradation caused by the roughness. Subsequently, only the effective region, except for the junction area between the ellipse and hyperbola, was covered with a thin chrome binder layer and 80-nm-thick rhodium using a homemade DC magnetron sputtering deposition system.

### Data processing of the obtained images

Sample images were obtained several times with an exposure of 10 s. The averaged image was processed by flat-field correction, in which the sample image was normalized with an empty image obtained without samples at the same condition. This was performed to correct the non-uniform illumination and uneven camera sensitivity distribution. Subsequently, the intrinsic mismatched magnifications of the advanced KB mirror optics between the vertical and horizontal directions were corrected. By digitally stretching the vertical direction by 3.25 fold, which corresponded to the designed value, the exact circle of the Siemens star successfully appeared, meaning that the experimentally obtained magnification mismatch was consistent with the designed one.

In the XAFS imaging, slight sample drift occurred on account of the long measurement time. The drift was corrected with sub-pixel accuracy by the template matching technique based on the normalized correlation coefficient.

### Contrast analysis

The image modulation was calculated from the corrected images according to the formula (*I*_*max*_(*r,θ*) − *I*_*min*_(*r,θ*))/*I*_*max*_(*r,θ*), where *I*_*max*_(*r,θ*) and *I*_*min*_(*r,θ*) represent the maximum and minimum, respectively, of the modulated intensity, and *r* and *θ* are the radius from the centre of the pattern and the azimuthal angle measured from the horizontal direction, respectively. The modulations were normalized by the modulation at the measured maximum feature of 1.5 μm as an ideal modulation given by the 500-nm-thick tantalum. To characterize the vertical and horizontal imaging performances, averaged modulations at *θ* = 0 and 180 degrees, and at *θ* = 90 and 270 degrees, respectively, were plotted in Fig. 4(c). A threshold modulation was set at 0.265, corresponding to the modulation at the Rayleigh criterion for a circular aperture^23^. Thus, the minimum spatial resolution was determined to be the smallest half-period that could be resolved with an image modulation of 0.265. Note that another formula exists for determining the image modulation: (*I*_*max*_(*r,θ*) − *I*_*min*_(*r,θ*))/(*I*_*max*_(*r,θ*) + *I*_*min*_(*r,θ*)). The corresponding threshold modulation is 0.153. Both formulas provide almost the same resolution, which is connected to the Rayleigh criterion (for more details, see ref. 23).

### Power spectrum analysis

PSA is often used to estimate the smallest detectable features that do not correspond with the Rayleigh limit^23^,^24^. It is easier to use and more robust for various noises than the contrast analysis. The analysis was performed as follows. First, the centred area of 2048 × 2048 pixels was trimmed and multiplied by the Hanning window function. It was subjected to the Fourier transform, and then the power spectrum was integrated azimuthally over 2π. A power spectrum without samples was calculated as follows. The empty images obtained for the flat-field correction were divided into two groups. The averaged image of the first group was normalized with the averaged one of the second group. The empty normalized image was processed in the same way. The one-dimensional power spectrum of the target image was plotted in the logarithmic scale together with the one of the empty images. The cut-off spatial frequency was determined from the intersected point with the empty image profile.

## Additional Information

**How to cite this article:** Matsuyama, S. *et al*. 50-nm-resolution full-field X-ray microscope without chromatic aberration using total-reflection imaging mirrors. *Sci. Rep.* **7**, 46358; doi: 10.1038/srep46358 (2017).

**Publisher's note:** Springer Nature remains neutral with regard to jurisdictional claims in published maps and institutional affiliations.

## Supplementary Material

Supplemental Materials

## Figures and Tables

**Table 1:** Parameters of the developed advanced KB mirror optics.

	H-Vert.	E-Vert.	H-Hori.	E-Hori.
	Vertical imaging		Horizontal imaging	
Shape	Hyperbola	Ellipse	Hyperbola	Ellipse
a (m)*	7.297 × 10^−2^	22.67	2.051 × 10^−2^	22.57
b (m)*	1.101 × 10^−3^	2.444 × 10^−2^	3.111 × 10^−4^	1.356 × 10^−2^
Incident glancing angle (mrad)**	4.67	5.51	4.73	5.51
Distance from object (mm)***	173	294	49.6	94.1
Mirror area length (mm)	100	120	30	39
Magnification factor	196		637	
Numerical aperture ( × 10^−3^)	1.44		1.51	

^*^Ellipse x^2^/a^2^ + y^2^/b^2^ = 1 or hyperbola x^2^/a^2^ − y^2^/b^2^ = 1.

^**^Averaged over the whole mirror area.

^***^At the centre of the mirror area.
