# Supplementary material for: 50-nm-resolution full-field X-ray microscope without chromatic aberration using total-reflection imaging mirrors
Source: Sci Rep. 2017 Apr 13;7:46358. doi: 10.1038/srep46358 (PMC5390314; doi:10.1038/srep46358)
Supplement: Supplemental Materials [file srep46358-s1.pdf]

Supplemental materials for

50-nm-resolution full-field X-ray microscope without chromatic aberration using total-reflection imaging mirrors

Satoshi Matsuyama[a,*], Shuhei Yasuda[a], Jumpei Yamada[a], Hiromi Okada[b], Yoshiki Kohmura[c], Makina Yabashi[c], Tetsuya Ishikawa[c], and Kazuto Yamauchi[a,d]

[a] Department of Precision Science and Technology, Graduate School of Engineering, Osaka University, 2-1 Yamada-oka, Suita, Osaka 565-0871, Japan

[b] JTEC Corporation, 2-4-35, Saito-Yamabuki, Ibaraki, Osaka 567-0086, Japan

[c] RIKEN SPring-8 Center, 1-1-1 Kouto, Sayo, Hyogo 679-5198, Japan

[d] Center for Ultra-Precision Science and Technology, Graduate School of Engineering, Osaka University, 2-1 Yamada-oka, Suita, Osaka 565-0871, Japan

[*] Corresponding author; matsuyama@prec.eng.osaka-u.ac.jp

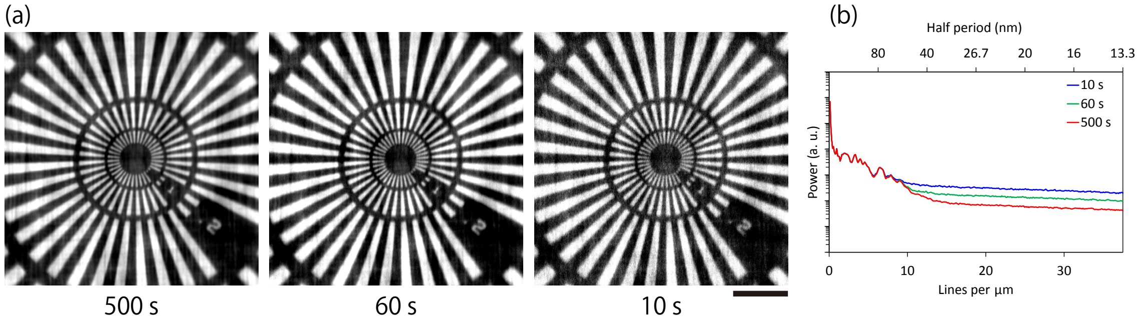

Supplemental Figure S1 (a) Bright-field X-ray images with exposures of 500 s, 60 s, and 10 s. (b) Result of PSA. The 500-s exposure image is the same as the image in Fig. 4. The images with exposures of 60 s and 10 s were produced using parts of the 500-s exposure image, which was produced by averaging 50 images with 10-s exposures. Even the 10-s-exposure image can resolve the 50-nm feature. The baselines in (b) show noise and a decrease with an exposure increase, following the Poisson noise rule. Bar = 2 μm.
